# Influence of Sulfur Induced Stress on Oxidative Status and Antioxidative Machinery in Leaves of *Allium cepa* L.

**DOI:** 10.1155/2014/568081

**Published:** 2014-10-29

**Authors:** Neelam Chandra, Nalini Pandey

**Affiliations:** Plant Nutrition and Stress Physiology Laboratory, Department of Botany, University of Lucknow, Lucknow 226007, India

## Abstract

A pot culture experiment was carried out to assess the effect of sulfur stress on growth, oxidative status, and antioxidative metabolism. Onion plants were treated with three different levels of sulfur, namely, 1.0, 4.0, and 8.0 mM S L^−1^. Plants raised with 4.0 mM S L^−1^ represent sufficient growth for the best vegetative yield. Plants supplied with 1.0 and 8.0 mM S L^−1^ showed retarded growth, chlorosis, and reduction in biomass and photoassimilatory pigments. Tissue sulfur concentration and cysteine were increased with increasing sulfur supply. Carbohydrates (sugars and starch) were accumulated in sulfur stressed plants. Hydrogen peroxide levels were increased in sulfur stressed plants. Thiobarbituric acid reactive substances levels were also increased which was an indicator of lipid peroxidation. Enzymatic (superoxide dismutase, catalase, peroxidase, ascorbate peroxidase, and glutathione reductase) and nonenzymatic (asorbate) antioxidative components were enhanced in sulfur stressed plants. Glutathione was increased with increasing sulfur supply. The present study showed that the adverse effects of inadequate sulfur supply result in irregular metabolic activities and antioxidant machinery.

## 1. Introduction

Recent studies have demonstrated that sulfur is important for the proper growth, metabolic activities, and development of plants. Sulfur is one of the most essential macronutrients required by the plants as it is an important constituent of amino acids such as cysteine and methionine and also in many metabolites [1].

Cysteine, as the first organic reduced sulfur compound, and methionine and its derivatives contribute to life not only as building blocks in proteins and their activity, but also as precursors for the synthesis of glutathione (GSH), cofactors (like Fe-S clusters, heme, siroheme, molybdenum centres, and lipoic acid), essential vitamins (biotin and thiamine), sulfur esters (coenzyme A), and sulfur derivatives. Sulfur is also an important constituent of several coenzymes, thioredoxins, and sulpholipids. Sulfur is also an important constituent of some compounds with may be involved in defense mechanisms against herbivores, pests, and pathogens or constituents to the special taste and odour of food plants [2]. Glutathione is a major thiol containing metabolite often present in millimolar concentrations and also associated with the defense system.

Sulfur is available primarily in the form of anionic sulfate (SO_4_^2−^) to plants which is transported through roots and then distributed via xylem to stem and other parts of plants [1]. Visible symptoms of sulfur deficiency such as chlorosis appear first in young leaves while older leaves remain green, suggesting that sulfur is immobile in older leaves. Insoluble sulfur deficient conditions [3]. Sulfur deficiency also affects CO_2_ assimilation rates and rubisco enzyme activities and protein abundance [4].

Reactive oxygen species (ROS) are generally produced during cellular metabolism and cells are always ready to cope up with this condition through their antioxidative machinery and scavenging enzymes. Nutrient deficiencies are also responsible for ROS production [5–7]. Chloroplast is an important source of producing ROS like superoxide (O_2_^−^) and hydrogen peroxide (H_2_O_2_) during reduced rate of photosynthetic carbon fixation [8]. Chloroplast can also produce ROS like singlet oxygen (^1^O_2_) through excited chlorophyll molecule [9]. Mitochondria and peroxisomes are also responsible for ROS production. In addition to these, ROS like hydroxyl radical (OH^∙^) can be formed from H_2_O_2_ and O_2_^∙^ through Haber-Weiss reaction [6].

The antioxidative machinery protects plants against this oxidative stress damage caused by ROS. Plants possess enzymatic (superoxide dismutase, catalase, peroxidise, ascorbate peroxidise, and glutathione reductase) and nonenzymatic (ascorbic acid and glutathione) components which work to protect the plant cells from oxidative damage by scavenging ROS [10]. So we have conducted this experiment in onion leaves, as sulfur has a very special role in onion plants, to evaluate changes in physiological and biochemical metabolism under induced sulfur stress. Sulfur has a marked effect on the pungency of the onion through increasing the pyruvic acid. The S-alkyl cysteine sulfoxides of the Alliaceae family contribute to the flavor and cancer prevention effects and generate S-containing volatile compounds upon tissue disruption and exposure to specific hydrolyzing enzymes [11]. When Allium species are grown with added sulfur, the increase in status of the plant correlates to an increase in the pungency [12]. When onion plants grown in the field were fertilized with sulfur, their S-propyl cysteine sulfoxides and S-methyl cysteine sulfoxide levels were more than double those of fertilized plants [13]. The level of S-alkyl cysteine sulfoxides is also dependent on sulfur nutrition [14].

## 2. Materials and Methods

Seeds of onion (*Allium cepa* L. var. Nasik-53) were sown in pots filled with purified and sterilized moist sand. Prior to sowing, the seeds were surface-sterilized with 5% (v/v) mercuric chloride solution and then with distilled water. The plants were treated with nutrient medium containing—4 mM KNO_3_, 4 mM Ca(NO_3_)_2_, 1.33 mM NaH_2_PO_4_, 0.33 *μ*M HBO_3_ 0.1 mM Fe EDTA, 10 *μ*M MnSO_4_, 1 *μ*M CuSO_4_, 1 *μ*M ZnSO_4_, 0.1 *μ*M Na_2_MoO_4_, 0.1 *μ*M NaCl, 0.1 *μ*M CoSO_4_, and 0.1 *μ*M NiSO_4_ [15]. Sulfur was supplied as MgSO_4_ and Na_2_SO_4_ at different levels. Sulfur was supplied as Na_2_SO_4_ and magnesium chloride is used for the compensation of magnesium in sulfur deficient plants. In sulfur excess plants, excess sulfur was supplied as Na_2_SO_4_. The experiment was conducted in randomized complete design with four replicates, and during the period in which the experiment was conducted, light intensity ranged between 605 to 745 *μ*mol m^−2^s^−1^ at noon. The temperature was ranged between 8–15°C and 17–38°C, and relative humidity ranged between 87 to 94% at 9:30 a.m. in glass house. The effects of three different sulfur applications (1.0, 0.4, and 8.0 mM S L^−1^) on growth, biomass, tissue sulfur, photoassmilatory pigments, cysteine, carbohydrates (sugars and starch), and antioxidative enzymatic and nonenzymatic components were evaluated, and the whole experiment was analysed at two stages 35 and 58 days of after treatment.

The plant material (leaves) was thoroughly cleaned by washing with tap water and then with deionzed water to remove surface contamination. After drying the fresh plant material in a forced drought oven at 70°C for 48 hours it was transferred to a desiccator and, when cool, was weighed accurately for the evaluation of biomass. Plant material (leaves) was wet-digested in nitric acid (HNO_3_) and perchloric acid (HClO_4_) which was in the ratio 0f 10 : 1 (V/V). After this, sulfur was evaluated turbidimetrically by the method of Chesnin and Yien [16]. Optical density was read within half an hour of the reaction at 430 nm on spectrophotometer.

For the estimation of photoassimilatory pigments, chopped leaves were ground in pestle mortar and extracted in 80% acetone with a pinch of calcium carbonate. Spectrophotometric measurements were made at 480 and 510 nm for carotenoids and 645 and 663 nm for chlorophylls [17]. To estimate sugar and starch in leaves, chopped leaves was fixed in boiling 80% ethanol. Sugars (reducing sugar and total sugar) were estimated colorimetrically by the method of Nelson [18] at 500 nm. Starch was estimated by the method of Montgomary [19]. Cysteine concentration was determined by the method of Gaitonde [20]. Fresh plant material (leaves) was homogenized in 5% chilled perchloric acid. Reaction mixture contained suitable amount of extract, glacial acetic acid, and ninhydrin reagent. Colour developed was read at 560 nm.

Hydrogen peroxide (H_2_O_2_) was estimated by the method of Brennan and Frenkel [21]. Thiobarbituric acid reactive substances (TBARS) were estimated by the method of Heath and Packer [22]. The absorbance was read at 532 nm and adjusted for nonspecific absorbance at 600 nm. The concentration of TBARS was estimated by using the extinction coefficient of 155 mM^−1^ cm^−1^. Ascorbate was determined according to the method of Law et al. [23] by extracting fresh leaf tissue in 10% TCA. The color was developed by 10% TCA, 44% orthophosphoric acid, 4% bipyridyl in 70% ethanol, and 3% ferric chloride and read at 525 nm. Glutathione was estimated by method of Ellman [24]. The reaction was carried out with the use of 10 mM DTNB and 0.1 mM GSH (glutathione reduced). The color intensity of extract was read out at 412 nm.

The activity of superoxide dismutase (SOD) was determined by measuring the ability to inhibit the photochemical reduction of nitro-blue tetrazolium (NBT). The reaction mixture contained 50 mM phosphate buffer pH 7.8, 13 mM methionine, 75 *μ*M NBT, 2 *μ*M riboflavin, 0.1 mM EDTA, and 0 to 50 *μ*L enzyme extract. Riboflavin was added at last and tubes were illuminated in sunlight for 10 min. Color density was read out at 560 nm. One unit of SOD represents the amount that inhibits the NBT reduction (Beauchamp and Fridovich) [25]. Catalase was assayed by the permagnate titration method of Euler and Josephson [26]. The enzyme assay was allowed to stand for 5 minutes at 25°C. The reaction mixture for enzyme assay contained 0.005 M hydrogen peroxide in 0.025 mM potassium phosphate buffer pH 7.0. The reaction was stopped by adding 2 N H_2_SO_4_. After stopping the reaction, titration was carried out. Peroxidase was assayed by the modified method of Luck [27]. The enzyme assay was carried out at 25°C in a solution containing 0.1 M phosphate buffer pH 6.0, 0.01% H_2_O_2_, and 0.5% p-phenyl diamine. The reaction was initiated by adding enzyme extract to the above and was allowed to proceed for 5 minutes. The reaction was stopped by adding 4 N H_2_SO_4_. The color intensity was read at 485 nm.

Activity of ascorbate peroxidase (APX) was determined as per the method of Nakano and Asada [28]. The reaction mixture consists of 50 mM potassium phosphate buffer pH 7.0, 0.5 mM ascorbate, and 0.1 mM hydrogen peroxide. Optical density was read out at 290 nm. The amount of ascorbate oxidized was calculated by using the extinction coefficient of 2.8 mM^−1 ^cm^−1^. Activity of glutathione reductase (GR) was evaluated by making a reaction mixture containing 100 mM phosphate buffer pH 7.0, 1 mM GSSG, 1 mM EDTA, 0.1 mM NADPH, and 25 to 50 *μ*L of the enzyme extract. The optical density was read out at 340 nm, and the amount of NADPH oxidized was calculated using the extinction coefficient of 6.22 mM^−1 ^cm^−1^ [29].

*Statistical Analysis*. The data has been presented in tables and figures and statistically evaluated by ANOVA. Differences between treatments means were compared using least significant differences [LSD at *P* ≤ 0.05] and as mean values standard error (±SE).

## 3. Result and Discussion

### 3.1. Plant Height, Growth, and Visible Morphology

Onion plants supplied with 1.0 mM S L^−1^ and 8.0 mM S L^−1^ showed an inhibition in growth leading to significant reductions in plant height [30] to the plant grown at 4.0 mM S L^−1^ which showed best vegetative growth and was treated as normal plants (Table 1). Highest reduction in plant height was observed in plants supplied with 8 mM S L^−1^ at 35 DAT; while at 58 DAT, there is not any difference in plant height of both S-deficient and S-excess plants. Plant growth was also reduced in sulfur deficient and excess plants. The deficiency symptoms of sulfur first appeared in young leaves. These were chlorosis and reduction in size of leaves. A mild chlorosis was observed at 20 DAT at the apex of leaves [30–32]. After 35 days chlorosis was spreading from apex to base of the leaves. At 35 days, tip burning was also observed in leaves of sulfur deficient and excess plants. Sulfur deficiency was also responsible for curling in leaves at later stage.

### 3.2. Biomass, Tissue S Concentration, and Cysteine

Biomass was reduced in sulfur stressed plants and reduction was more prominent in S-excess plants. Tissue S concentration and cysteine were increasing with increasing S supply from 1.0 to 8.0 mM S L^−1^ (Table 1) which is in consonance with the results of Chandra and Pandey [30, 32]. It is generally well known that the uptake and assimilation of sulfate is induced under conditions of sulfur starvation or high demand, for sulfur metabolites. Sulfate is incorporated into the soluble fraction of leaves which are about 70% expanded and most of this sulfur is redirected into new expanding leaves [33, 34]. Sulfur stress does not increase the export of sulfur from mature leaves to Young leaves as reported earlier by Adiputra and Anderson [35]. Export of sulfur from the leaves of sulfur deficient plants was restricted to the export of sulfur from the soluble fraction, as in normal plants. In S-deficient plants, young recipient leaves incorporate less sulfur from the soluble fraction into the soluble fraction. Due to these reasons, sulfur deficiency symptoms occur in youngest leaves of S-deficient plants [36].

Transportation of sulfur in plants is carried out by a complex system of transporters encoded by a large gene family [37]. In leaves, AtSultr 2 : 1 is expressed in the xylem parenchyma and phloem cells; but in the root it is expressed in xylem parenchyma and pericycle cells. In leaves, a considerable diversity of patterns of expression may be visualized during development and in response to fluctuating sulfur availability. Group 3 consists of 5 sulfate transporters (AtSultr 3 : 1, 3 : 2, 3 : 3, 3 : 4, and 3 : 5) and are known as leaf expressed group [38]. Formation of cysteine was directly related to S concentration as it contains S. Cysteine plays a very important role in plant metabolism as it regulates glutathione synthesis. Cysteine also functions as a sulfur donor for methionine and secondary metabolites biosynthesis [1]. Cysteine in the low molecular weight glutathione (GSH) and derivatives (phytochelatin polymers) also plays a critical role in production against abiotic/biotic stress [39, 40]. Sulfur starvation is responsible for the increase of OAS (O-acetyl serine) levels, which in turn induces the expression of genes encoding sulfate transporters and APS reductase, thereby overriding the repressive effect of sulfur-sufficient nutritional conditions [41]. OAS (thiol-) lyase, catalyzing the reaction of formation of cysteine from OAS and hydrogen sulfide, is in the same subcellular compartments as serine acetyltransferase. OAS accumulation stimulated by S-deficiency further promotes the dissociation of the complex to reduce the activity of ser acetyltransferase, resulting in reduced OAS formation. In turn, upon excess sulfur supply, accumulated sulfide promotes formation of the complex leading to stimulated OAS formation to maintain cysteine synthesis [42].

### 3.3. Photoassimilatory Pigments

Chlorophyll (chl a and chl b) and carotenoid (car) reduced in S-stressed plants, and reduction was found to be more significant in S-deficient plants in comparison to S-excess plants. Highest reduction in chlorophyll (a and b) concentration was observed under S-deficiency. In comparison to 35 DAT, chl a, chl b, and car were reduced more at 58 DAT in S-stressed plants which shows that severe deficiency results in reduced chlorophyll (Table 2). Reduction in chlorophyll concentration was also observed by Lunde et al. [43], Brennan et al. [31], and Tewari et al. [44] in rice, canola, and mulberry, respectively. At 35 DAT ratio of chl a and b was increased while at 58 DAT it was increased with increasing sulfur supply. Carotenoid concentration was decreased more at 58 DAT in comparison to 35 DAT in S-stressed onion plants. The ratio of chlorophyll and carotenoid was decreased under sulfur stressed plants in comparison to normal plants (Table 2).

### 3.4. Carbohydrates

Sulfur concentration also alters carbohydrates concentration in the leaves of onion plants. Sugars and starch both were accumulated in S-stressed plants. Reducing sugar accumulated more in S-deficient plants while nonreducing sugar was higher in S-excess plants. Total sugar was accumulated in sulfur stressed plants and more prominent in leaves of S-deficient plants and especially at 58 DAT. Starch also showed same trend as total sugar (Table 3). Starch accumulation under sulfur deficiency was also observed by Lunde et al. [43].

Reduced chlorophyll and decreased rubisco concentration was observed under sulfur deficiency [45]. Starch accumulation in S-deficient plants was better described on the basis of Fd:thioredoxin system [46, 47]. Carbohydrate concentration is directly dependent on photosynthetic process. Photosynthesis is essentially a redox process and it makes substantial use of sulfur. Sulfur atoms are located in compartments of the electron transfer chain and in carriers of reducing power generated by the photosynthetic electron transfer (e.g., ferredoxin). Disulfide bridges also play an important role in carbohydrate metabolism as they are involved in light/dark regulation of Calvin cycle enzymes [48]. There are few reports that excess S in form of sulfate is responsible for carbohydrate accumulation. But excess sulfur in form of SO_2_ was responsible for the accumulation of carbohydrate [49].

### 3.5. H_2_O_2_, TBARS, ASC, and GSH

**Figure 1:**
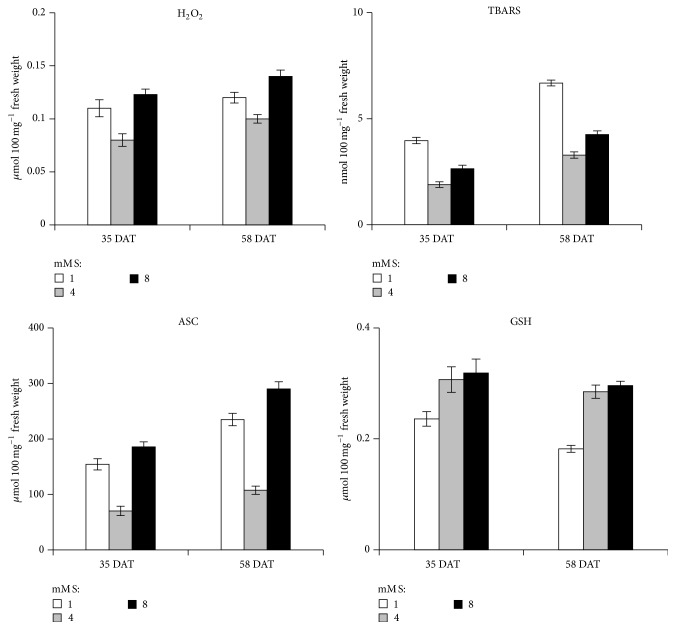
Effect of sulfur supply on hydrogen peroxide (H_2_O_2_), thiobarbutric acid reactive substances (TBARS), ascorbate (ASC), and glutathione (GSH) in leaves of onion (*Allium cepa* L. var. Nasik-53) plants (levels mM S L^−1^: 1.0, 4.0, and 8.0). Data represent mean ± S.E. (*P* ≤ 0.05).

Increased accumulation of ROS in plant tissues is an oxidative common condition in stressed plants. Under stress, plants redox equilibrium is altered and ROS accumulation causes specific oxidative stress. Compared with the control plants, S-deficient and excess conditions significantly increased the concentration of H_2_O_2_ (Figure 1). Increment in H_2_O_2_ due to sulfur deficiency was reported by Tewari et al. [44, 50] in mulberry and maize respectively. At 35 DAT, H_2_O_2_ concentration was increased up to 38% and 54% in 1 and 8 mM S L^−1^ by normal plants. But at 58 DAT, H_2_O_2_ concentrations were increased as 23% and 40% in 1 and 8 mM S L^−1^ as compared to normal plants. This shows that antioxidants were trying to remove the excess of H_2_O_2_ molecule and minimizing the oxidative damage. Excess H_2_O_2_ can be transferred via the Haber-Weiss reaction to form highly reactive oxidant OH which leads to the lipid peroxidation. In the present study, concentration of TBARS which was found to be increased in stressed plants often indicates severe lipid peroxidation. Concentration of TBARS showed a significant increase at 58 DAT which shows severe stressed condition (Figure 1). Percentage increase was found to be highest (110%) at 35 DAT in plants supplied with 1 mM s L^−1^. The present investigation revealed that the concentration of ascorbate was increased in both S-deficient and S-excess supplied plants in comparison to normal plants (Figure 1). Concentration of glutathione was increased with increasing sulfur supply. Our results were in consonance with the reports of Chandra and Pandey [32]. Ascorbate concentration was more at 8 mM S L^−1^ in comparison to 1 mM S L^−1^. At 58 DAT, percentage increase (171%) was the highest at 8 mM S L^−1^. Ascorbate is the powerful antioxidant which helps the plant metabolism to minimizing the damage caused by different reactive oxygen species. Chloroplast contains about 30 to 40% of total ascorbate [51] and, except for scavenging ROS, it has also a special role in preserving the activities of different enzymes that contain prosthetic transition metal ions [52]. Glutathione is an important S containing metabolite found in plants which prevents ROS induced oxidative damage. It also plays an essential role in the regulation of sulphate transport, signal transduction, conjugation of different metabolite, and detoxification of xenobiotics [53]. Glutathione is also important to maintain reduced state of cells to counteract the inhibitory effects of oxidative stress [54]. Concentration of GSH is totally dependent upon the sulfate availability. In present experiment concentration of GSH was increased with increasing S supply (Figure 1). Glutathione is also thought to be a phloem-translocated signal molecule that represses the genes of sulfur assimilation [55].

### 3.6. Antioxidative Enzymes

**Figure 2:**
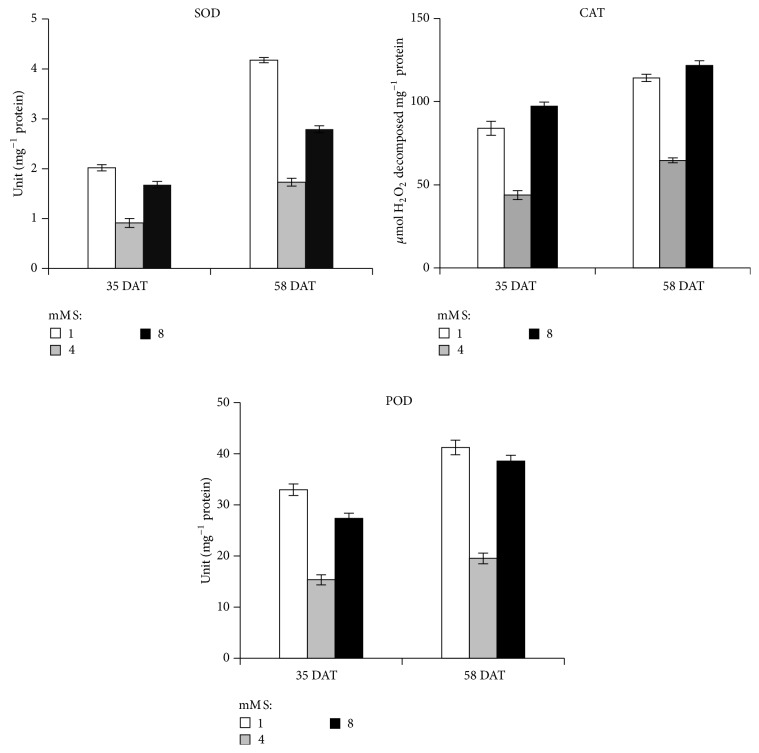
Effect of sulfur supply on superoxide dismutase (SOD), catalase (CAT), and peroxidase (POD) in leaves of onion (*Allium cepa* L. var. Nasik-53) plants (levels mM S L^−1^: 1.0, 4.0, and 8.0). Data represent mean ± S.E. (*P* ≤ 0.05).

To cope with oxidative damage, plants have an efficient antioxidative mechanism. In this study, a significant increase in SOD activities in leaves was observed under sulfur deficiency which was in consonance with the reports of Tewari et al. [44, 50]. The activity of SOD was also increased in S-excess plants. Superoxide dismutase activity was very high in S-deficient plants in comparison to S-excess plants at both stages (Figure 2). SOD activity increases to convert superoxide radical to H_2_O_2_ which is also a ROS and damage membrane. And plants will be completely protected when H_2_O_2_ is converted into water and oxygen. H_2_O_2_ is efficiently removed by catalase and peroxidase. Increased activity of CAT and POD was observed at both stages in leaves of onion plants which may remove excess H_2_O_2_ caused by S-stress and thus play a detoxifying role. Percentage increase in activity was found to be more at 35 DAT in comparison to 58 DAT, while CAT activity was more at 58 DAT. Peroxidase activity was observed more at 58 DAT in comparison to plants analyzed at 35 DAT both under S-deficient and S-excess plants (Figure 2).

**Figure 3:**
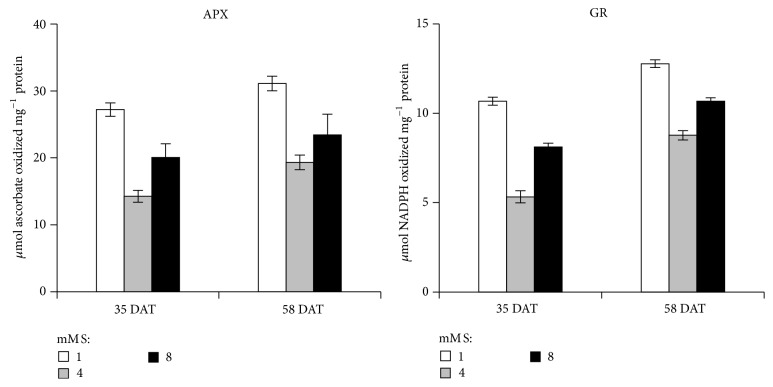
Effect of sulfur supply on ascorbate peroxidase (APX) and glutathione reductase (GR) in leaves of onion (*Allium cepa* L. var. Nasik-53) plants (levels mM S L^−1^: 1.0, 4.0, and 8.0). Data represent mean ± S.E. (*P* ≤ 0.05).

Activity of APX and GR was also found to be more at 58 DAT in comparison to 35 DAT (Figure 3). The highest activity of APX and GR was observed at 1 mM S L^−1^ at 58 DAT. APX has a very special role in scavenging ROS through water-water and ASH-GSH cycles by utilizing ASH as the electron donor. And GR is an enzyme of ASH-GSH cycle. Glutathione reductase also detoxifies plant cells by scavenging ROS by sustaining the reduced status of GSH. It catalyzes the reduction of glutathione and also catalyzes the NADPH-dependent reaction of disulphide bond of GSSG [10].

In conclusion, onion leaves treated with low- and high-S induced a concentration-dependent oxidative stress characterized by accumulation of hydrogen peroxide with increased lipid peroxidation levels, increased superoxide dismutase, catalase, peroxidase, ascorbate peroxidise, and glutathione reductase displaying a better antioxidant response. Ascorbate was also increased, and glutathione was increased with increasing sulfur supply. Finally, current study identified the potential role of sulfur in metabolism of onion plants. Ascorbate was also increased and glutathione was increased with increasing sulfur supply. Finally, current study identified the potential role of sulfur in metabolism of onion plants.

## Figures and Tables

**Table 1:** Effect of variable sulfur supply on plant height, biomass and tissue sulfur concentration in leaves of onion plants (*Allium cepa* L. var. Nasik-53) at 35 and 58 days after treatment.

Days after treatment	Plant parts	mM S L^−1^ supply		
		1.0	4.0	8.0
Plant height: cm				
35		15.89 ± 1.101^b^	18.33 ± 1.208^a^	13.24 ± 1.015^b^
58		26.37 ± 2.203^b^	30.75 ± 2.031^a^	26.89 ± 2.011^b^

Biomass: g plant^−1^				
35	Leaves	0.072 ± 0.008^b^	0.089 ± 0.004^a^	0.064 ± 0.004^b^
58	Leaves	0.137 ± 0.011^b^	0.153 ± 0.013^a^	0.116 ± 0.018^c^

Sulfur: % dry weight				
35	Leaves	0.104 ± 0.012^a^	0.212 ± 0.010^b^	0.378 ± 0.015^c^
58	Leaves	0.232 ± 0.026^a^	0.442 ± 0.029^b^	0.502 ± 0.038^c^

Values are mean ± SE, *n* = 4. Data with different superscript letters in the same row indicate a significant difference at *P* ≤ 0.05.

**Table 2:** Effect of variable sulfur supply on photoassimilatory pigments (chlorophyll a and b and total chlorophyll and carotenoid) in leaves of onion (*Allium cepa* L. var. Nasik-53) plants at 35 and 58 days after treatment.

Days after treatment		mM S L^−1^ supply		
		1.0	4.0	8.0
Chlorophyll: mg g^−1^ fresh weight				
35	Chl a	0.432 ± 0.013^a^	0.803 ± 0.017^c^	0.522 ± 0.019^b^
	Chl b	0.211 ± 0.024^b^	0.447 ± 0.018^a^	0.242 ± 0.027^b^
	Total chl	0.643 ± 0.031^a^	1.250 ± 0.044^b^	0.764 ± 0.053^c^
	Chl a/b	2.047 ± 0.151^a^	1.796 ± 0.160^ b^	2.157 ± 0.158^c^
58	Chl a	0.209 ± 0.011^a^	0.615 ± 0.021^c^	0.331 ± 0.016^b^
	Chl b	0.167 ± 0.014^a^	0.376 ± 0.023^c^	0.190 ± 0.022^b^
	Total chl	0.376 ± 0.034^a^	0.991 ± 0.039^c^	0.521 ± 0.047^b^
	Chl a/b	1.251 ± 0.103^a^	1.635 ± 0.118^b^	1.742 ± 0.112^c^

Carotenoids: mg g^−1^ fresh weight				
35	Car	0.362 ± 0.012^b^	0.482 ± 0.014^a^	0.397 ± 0.015^c^
	Chl/car	1.776 ± 0.178^b^	2.593 ± 0.172^a^	1.924 ± 0.168^b^
58	Car	0.211 ± 0.017^a^	0.374 ± 0.016^c^	0.230 ± 0.021^b^
	Chl/car	1.781 ± 0.163^a^	2.649 ± 0.182^b^	2.265 ± 0.160^c^

Values are mean ± SE, *n* = 4. Data with different superscript letters in the same row indicate a significant difference at *P* ≤ 0.05.

**Table 3:** Effect of variable sulfur supply on carbohydrates (reducing sugar, nonreducing sugar, and total sugar and starch) and cysteine concentration in leaves of onion plants (*Allium  cepa *L. var. Nasik-53) at 35 and 58 days after treatment.

Days after treatment		mM S L^−1^ supply		
		1.0	4.0	8.0
Carbohydrates: % fresh weight				
35	Reducing sugar	0.189 ± 0.013^b^	0.073 ± 0.017^a^	0.151 ± 0.012^c^
	Nonreducing sugar	0.040 ± 0.022^a^	0.053 ± 0.013^b^	0.067 ± 0.024^c^
	Total sugar	0.229 ± 0.027^b^	0.126 ± 0.030^a^	0.218 ± 0.031^b^
	Starch	0.913 ± 0.162^c^	0.704 ± 0.143^a^	0.822 ± 0.137^b^
58	Reducing sugar	0.252 ± 0.019^b^	0.130 ± 0.014^a^	0.276 ± 0.013^b^
	Nonreducing sugar	0.651 ± 0.031^c^	0.102 ± 0.027^a^	0.577 ± 0.021^b^
	Total sugar	0.903 ± 0.024^b^	0.232 ± 0.032^a^	0.853 ± 0.029^b^
	Starch	1.115 ± 0.124^c^	0.775 ± 0.136^a^	0.900 ± 0.147^b^

Cysteine: mM 100 mg^−1^				
35	Cysteine	0.164 ± 0.004^a^	0.183 ± 0.003^b^	0.227 ± 0.006^c^
58	Cysteine	0.122 ± 0.008^a^	0.166 ± 0.006^b^	0.187 ± 0.004^c^

Values are mean ± SE, *n* = 4. Data with different superscript letters in the same row indicate a significant difference at *P* ≤ 0.05.
